# A comparison of post-transplantation cyclophosphamide versus antithymocyte-globulin in patients with hematological malignancies undergoing HLA-matched unrelated donor transplantation

**DOI:** 10.1097/MD.0000000000021571

**Published:** 2020-08-21

**Authors:** Myung-Won Lee, Sang Hoon Yeon, Won-Hyoung Seo, Hyewon Ryu, Hyo-Jin Lee, Hwan-Jung Yun, Deog-Yeon Jo, Ik-Chan Song

**Affiliations:** a Department of Internal Medicine; b Center of Hematopoietic stem cell transplantation, Chungnam National University Hospital, South Korea.

**Keywords:** antithymocyte-globulin, cost, graft-versus-host disease, post-transplantation cyclophosphamide

## Abstract

Post-transplantation cyclophosphamide (PTCy) and antithymocyte-globulin (ATG) are the most commonly used regimens for prophylaxis of graft-versus-host disease (GVHD). We compared these 2 regimens in human leukocyte antigen (HLA)-matched unrelated donor hematopoietic stem cell transplantation (HSCT) patients with hematological malignancies. We retrospectively analyzed consecutive adult patients with hematological malignancies who underwent HLA-matched unrelated donor-HSCT at Chungnam National University Hospital (Daejeon, South Korea) between January 2013 and January 2019. Patients who received a second transplantation or who had refractory disease were excluded. We included 34 patients (12 and 22 in the PTCy and ATG groups respectively). All graft sources were peripheral blood stem cells. The estimated 20-month overall survival rates were 75.0% for PTCy and 81.6% for ATG patients (*P* = .792), and the 20-month relapse rates were 41.7% and 34.3% (*P* = .491), respectively. The cumulative incidences of grade 2 to 4 acute GVHD were 16.7% and 30.6% (*P* = .551), respectively; the estimated 20-month limited and extensive chronic GVHD rates were 59.1% and 78.8% (*P* = .718), respectively; and the estimated 20-month extensive chronic GVHD rates were 12.5% and 16.7% (*P* = .718), respectively. The neutrophil engraftment time was similar in both groups [median (range), 15.0 (12.0–17.0) and 14.0 (12.0–19.0) days, respectively; *P* = .961]. However, ATG was more expensive than PTCy [median (range), US$4,062 (US$2,215–6,647) for ATG vs US$51.80 (US$43.20–69.20) for PTCy; *P* < .001]. In conclusion, PTCy and ATG afforded similar clinical outcomes after HLA-matched unrelated donor transplantation but PTCy was less expensive.

## 1 Introduction

Allogeneic hematopoietic stem cell transplantation (HSCT) is a valuable curative therapy for hematological malignancies; however, graft-versus-host disease (GVHD) remains of concern, although recent advances have reduced its incidence.^[1]^ Post-transplantation cyclophosphamide (PTCy) is often used to prevent GVHD, especially in patients who have undergone haplo-identical transplantation.^[2,3]^ PTCy effectively eradicates allo-reactive T cells after haplo-identical stem cell infusion, but hematopoietic stem cells are spared; the drug thus reduces the incidences of acute and chronic GVHD.^[4,5]^ PTCy is now one of the standard regimen of GVHD prophylaxis after haplo-identical HSCT.^[6]^ Antithymocyte-globulin (ATG) is widely used for GVHD prophylaxis in patients undergoing human leukocyte antigen (HLA)-matched donor HSCT, significantly enhancing overall survival and GVHD-free survival compared to those of patients not given ATG.^[7,8]^ PTCy has also been tested in the HLA-matched donor HSCT setting. PTCy usefully prevented GVHD of HLA-matched sibling or unrelated donor transplant patients.^[9,10]^ However, very few comparisons of PTCy and ATG in terms of GVHD prophylaxis and the clinical outcomes of HLA-matched unrelated donor HSCT have appeared. The current study aimed to compare these 2 approaches for GVHD prophylaxis in patients with hematologic malignancies undergoing HLA-matched unrelated donor transplantation.

## 2 Materials and methods

### 2.1 Patients and treatments

We retrospectively analyzed consecutive adult patients (age, >18 years) with hematological malignancies who underwent HLA-matched unrelated donor allogeneic HSCT in Chungnam National University Hospital (Daejeon, South Korea) between January 2013 and January 2019. We excluded those receiving second transplantations and patients with refractory disease. PTCy was given on days +3 and +4 at a dose of 50 mg/kg. Rabbit ATG (thymoglobulin; Sanofi-Aventis, Paris, France) was given from days -4 to -2 at a dose of 1.5 mg/kg. We randomly assigned PTCy and ATG for prophylaxis of GVHD. Two conditioning regimens were used. In the myeloablating conditioning (MAC) regimen, 3.2 mg/kg busulfan was administered for 4 days and 40 mg/m^2^ fludarabine was administered for 5 days. In the reduced intensity conditioning (RIC) regimen, 3.2 mg/kg busulfan was administered for 2 days and 30 mg/m^2^ fludarabine was administered for 6 days. RIC was administered to patients over 55 years of age or with comorbidities. No pharmacokinetic adjustment of busulfan dose was performed. Tacrolimus for GVHD prophylaxis was given commencing on day –1 with a target level of 5 to 15 ng/mL. All patients received granulocyte colony-stimulating factor-mobilized peripheral blood stem cells (PBSCs; target CD34+ cell count, 5 x 10^6^/kg). Filgrastim 5ug/kg was administered from day +5 until neutrophil recovery. Any therapy for prevention of relapse after allogeneic HSCT, such as donor lymphocyte infusion or hypomethylating agents or tyrosine kinase inhibitors, was not added.

### 2.2 Outcomes

The primary outcome was overall survival, and the secondary outcomes were the incidences and severity of acute and chronic GVHD, leukemia-free survival, the relapse rate, non-relapse mortality (NRM), the neutrophil and platelet engraftment times, cytomegalovirus (CMV), and EBV reactivation, days of hospitalization and cost-effectiveness. Acute GVHD was graded using modified Seattle Glucksberg criteria and chronic GVHD by reference to the revised Seattle criteria.^[11,12]^ NRM was defined as death from any cause other than relapse. CMV and EBV reactivation was defined as detection of viral DNA in whole blood by PCR at least once. Cardiotoxicity was evaluated by echocardiography performed at 3 months after HSCT.

### 2.3 Statistical analysis

Categorical variables were compared using the chi-squared test and logistic regression was employed to examine correlations. Overall and leukemia-free survival was assessed using the Kaplan-Meier method. Survival rates were compared using the log-rank test. Cumulative incidence functions were used to estimate the acute and chronic GVHD rates, relapse rate, and NRM. A *P*-value <.05 was considered to reflect significance. All statistical analyses were performed with the aid of SPSS software ver. 24.0 (IBM Corporation, Armonk, NY).

### 2.4 Ethics statement

The study protocol was approved by the Institutional Review Board of Chungnam National University Hospital. The need for informed patient consent was waived given the retrospective nature of the analysis.

## 3 Results

### 3.1 Patient characteristics

In total, 34 patients were enrolled (12 in the PTCy group and 22 in the ATG group, with median ages of 56 and 48.5 years respectively). The most common condition was acute myeloid leukemia (AML) followed by acute lymphoblastic leukemia (ALL) and myelodysplastic syndrome. Disease status at transplant was similar in both groups. Poor risked patients, who included secondary AML, therapy-related AML or had poor cytogenetics in NCCN guidelines, were assigned equally to both groups. All patients underwent mobilized, peripheral blood HSCT. Fifty percent of the PTCy group patients received the MAC regimen, as did 68.2% of the ATG group patients. The follow-up duration was longer in the ATG group than in the PTCy group [40(range, 3–60) and 19 (range, 3–34) months respectively; *P* = .006]. Table 1 summarizes the patient demographics.

**Table 1:**
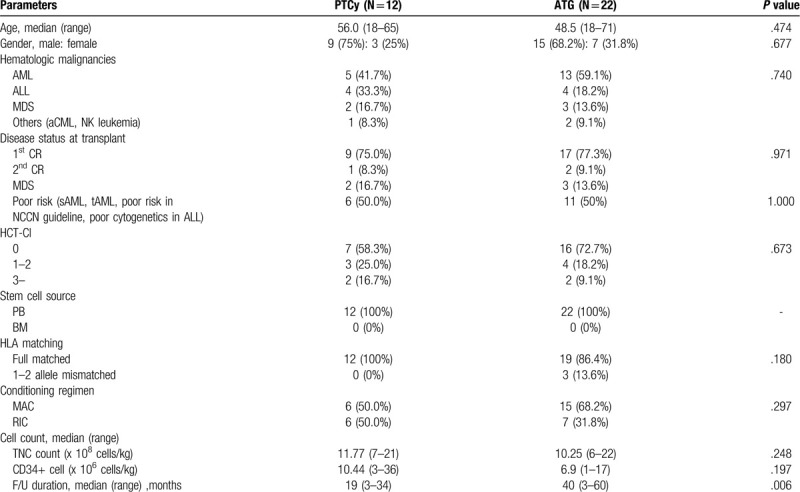
Patients’ demographics (N = 34).

### 3.2 Survival outcomes

**Figure 1:**
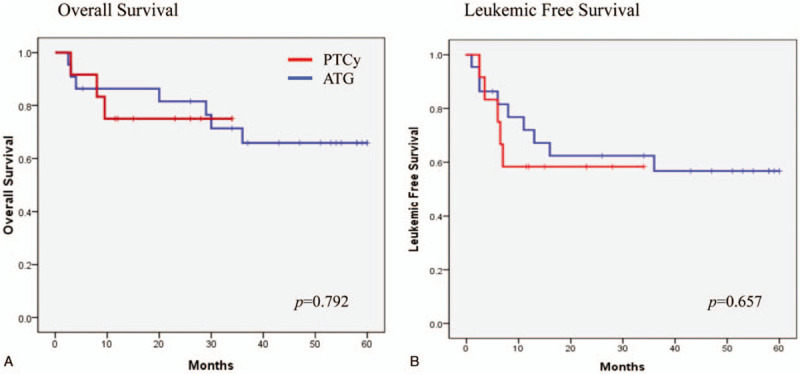
(A) Overall survival and (B) Leukemic free survival.

**Figure 2:**
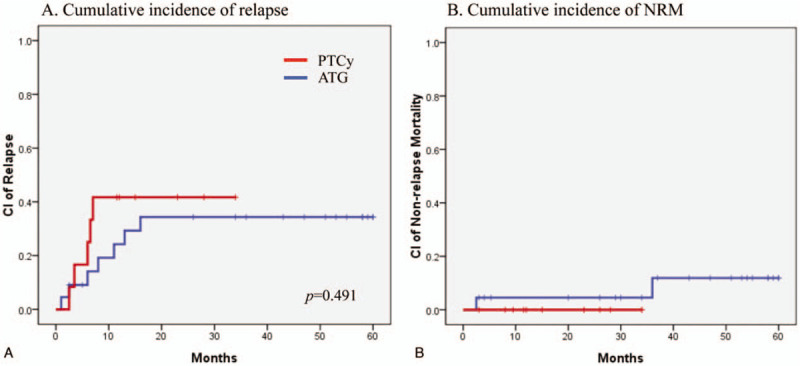
(A) Cumulative incidence of relapse and (B) Non-relapse mortality.

The median overall survival was not attained by either group (Fig. 1A). The 20-month survival rates were 75.0% in the PTCy group and 81.6% in the ATG group (*P* = .792). The 20-month leukemia-free survival rates were 58.3% in the PTCy group and 62.4% in the ATG group (*P* = .657, Fig. 1B). The cumulative 20-month relapse rates were 41.7% in the PTCy group and 34.3% in the ATG group (*P* = .491, Fig. 2A). The cumulative 20-month NRM incidence was 0% in the PTCy group and 4.5% in the ATG group (*P* = .460, Fig. 2B).

### 3.3 GVHD

**Figure 3:**
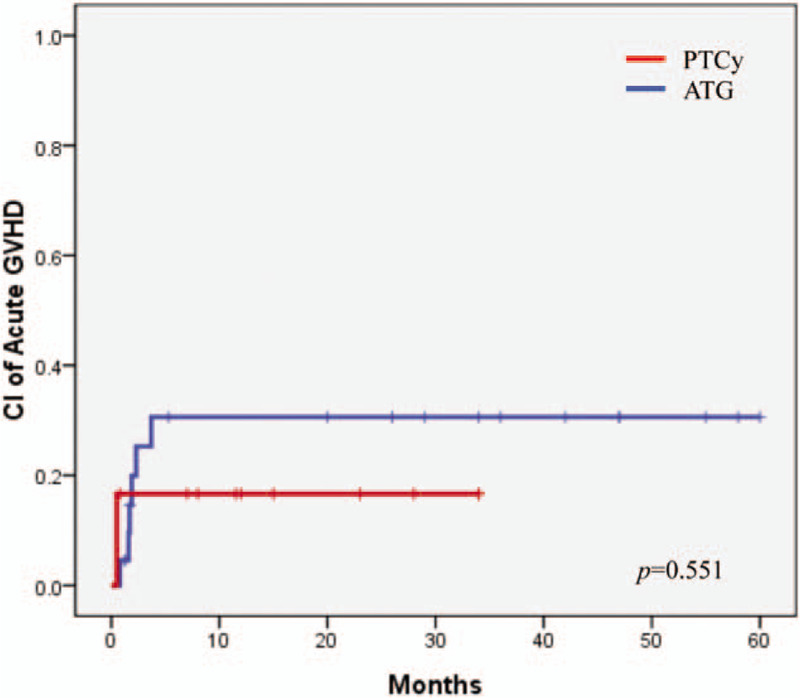
Cumulative incidence of acute GVHD, grade 2 to 4.

**Figure 4:**
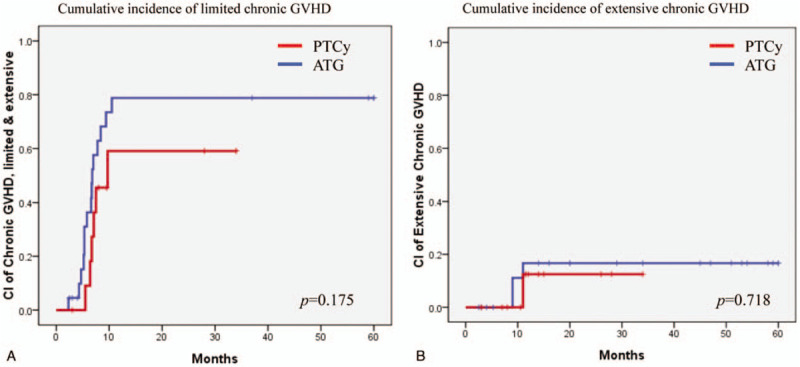
(A) Cumulative incidence of limited chronic GVHD and (B) extensive chronic GVHD.

No patient exhibited acute GVHD of grade 3 or higher. The cumulative incidence of grade 2 acute GVHD was 16.7% in the PTCy group and 30.6% in the ATG group (*P* = .551, Fig. 3). The cumulative 20-month incidence of limited chronic GVHD was 59.1% in the PTCy group and 78.8% in the ATG group (*P* = .175, Fig. 4A). The cumulative 20-month incidence of extensive chronic GVHD was 12.5% in the PTCy group and 16.7% in the ATG group (*P* = .718, Fig. 4B).

### 3.4 Other secondary outcomes

The median neutrophil engraftment times were 15 (range, 12–17) and 14 (range, 12–19) days in the PTCy and ATG groups, respectively (*P* = .961). The median platelet engraftment times were 13 (range, 12–54) and 13 (range, 10–40) days, respectively (*P* = .130). The cytomegalovirus reactivation rates were 41.7% and 31.8%, respectively (*P* = .566). The EBV reactivation rates were 8.3% and 9.1%, respectively (*P* = .941). And there were no post-transplant lymphoproliferative disease and cardiotoxicities in either group. The rates of hepatic veno-occlusive disease were 8.3% and 9.1%, respectively (*P* = .941). The rates of BK virus-associated hemorrhagic cystitis were 0% and 4.5%, respectively (*P* = .453). The median days of hospitalization were 34 (range, 30–43) days and 34 (range, 28–93) days, respectively (*P* = .245). The median costs of the PTCy and ATG treatments were US$51.80 (range, US$43.20–69.20) and US$4062 (range, US$2215–6647) (*P* < .001). Table 2 summarizes the other secondary outcomes.

**Table 2:**
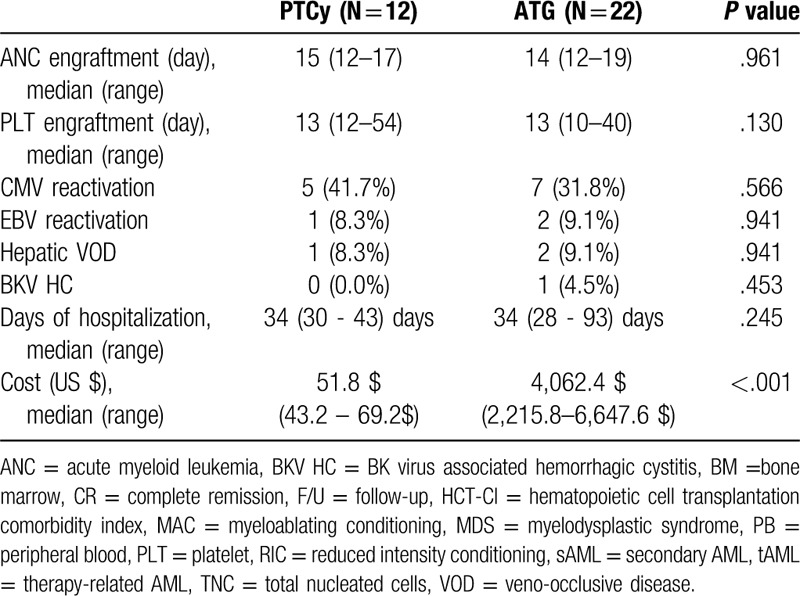
Other secondary outcomes.

## 4 Discussion

We found that PTCy and ATG afforded similar survival outcomes and controlled GVHD incidence and severity equally well. PTCy has been used mainly in haplo-identical HSCT patients until now.^[13,14]^ The study of EBMT registry reveals that PTCy-treated patients had significantly fewer incidences of grade 3 to 4 acute GVHD compared to ATG-treated patients, and PTCy is associated with better overall survival, GVHD-free, and leukemia-free survival than is ATG in haplo-identical transplant.^[6]^ We found that the relapse rate was slightly higher in the PTCy than in the ATG group in this study. However, this remains controversial, as no direct comparison of PTCy and ATG in terms of relapse rate in HLA-matched unrelated donor transplant has been published. Some authors consider that the strong immunosuppressive effects of PTCy reduce not only GVHD incidence but also anti-tumor activity.^[15,16]^ However, a clinical trial with a large number of patients showed that patients receiving PTCy and ATG had similar relapse rates.^[6]^

Ruggeri et al evaluated EBMT data in terms of PTCy-mediated GVHD prophylaxis in HLA-matched sibling and unrelated donor transplant patients and found that the addition of immunosuppressive drugs to PTCy was associated with a reduced risk of extensive chronic GVHD and increased overall survival.^[10]^ The cumulative 2-year relapse incidence was 36% when PTCy was combined with 1 immunosuppressive drug, similar to what we found. Ruggeri et al found that the incidences of extensive chronic GVHD were 18% in those given PTCy alone, and 20% or 8.5% in patients given PTCy and 1 or 2 immunosuppressive drugs, respectively. Because PTCy effectively suppresses GVHD, when 2 immunosuppressive agents are added, graft-versus-leukemic effect may be weakened, which increases the risk of relapse, so we added only tacrolimus to PTCy in this study; the cumulative 20-month incidence of extensive chronic GVHD was 12.5%, thus quite similar to the EBMT registry data although we used peripheral blood as the stem cell source for all patients. The EBMT registry and previous reports contain data on quite a large number of patients who underwent bone-marrow HSCT; 60% of EBMT haplo-identical transplantation patients were in this category.^[6]^ About 25% patients of EBMT registry who underwent HLA-matched sibling and unrelated donor transplantations received bone marrow hematopoietic stem cells.^[10]^ In the work of Bashey, about 60% of patients underwent bone-marrow haplo-identical transplantation.^[2]^ Generally, mobilized, peripheral blood stem cell transplantation is associated with more severe GVHD than bone marrow transplantation.^[17,18]^ However, we found that PTCy effectively prevented chronic GVHD even after mobilized peripheral blood stem cell transplantation. Mielcarek et al also reported comparable clinical outcomes using PTCy for GVHD prophylaxis after HLA-matched mobilized blood cell transplantation.^[19]^ In terms of cost-effectiveness, ATG is about 80-fold more expensive than PTCy in South Korea. The neutrophil and platelet engraftment times, and the hospitalization times, were similar in both groups. As HSCT requires a long duration of hospitalization and is thus expensive, PTCy could reduce the economic burdens imposed on patients and the national health insurance system.^[14,20]^

ATG was generally administered from day -3 to -1 prior to hematopoietic stem cell infusion, but in this study we used ATG from day -4 to -2 before stem cell infusion. However, since the half-life of ATG is as long as 30 days and is detected even up to 5 weeks after HSCT, it is thought that the ATG schedule has little effect on GVHD.^[21,22]^ In the ATG group of our study, 3 patients underwent 1 or 2 allele mismatched unrelated donor transplantation of 10 HLAs, but these patients were inconsistent with HLA-DRB1∗11:01–11:04 and HLA-DQB1∗03:01–03:02 or HLA-C∗03:03–03:04 alleles. Since these allele mismatches was considered to be permissible for HSCT, it is thought that these HLA mismatch had little effect on GVHD.^[23,24]^ In addition, patients in the ATG group tended to received myeloablating conditioning therapy more frequently than patients in the PTCy group. This is a limitation that occurs because this study is a retrospective study.

To the best of our knowledge, this is the first study to directly compare PTCy and ATG for HLA-matched, unrelated donor transplantation patients. Although our patient numbers were small, we enrolled consecutive patients treated with identical protocols in a single institute. Thus, our data are relevant to real-world practice. In conclusion, PTCy appears to be a safe and inexpensive method of GVHD prophylaxis compared to ATG in HLA-matched unrelated donor HSCT.

## Author contributions

**Conceptualization:** Myung-Won Lee, Ik Chan Song.

**Data curation:** Myung-Won Lee, Sang-Hoon Yeon.

**Formal analysis:** Myung-Won Lee, Won-Hyoung Seo.

**Funding acquisition:** Ik Chan Song.

**Investigation:** Myung-Won Lee, Hyewon Ryu, Hyo-Jin Lee, Hwan-Jung Yun, Deog-Yeon Jo.

**Supervision:** Ik Chan Song.

**Writing – original draft:** Myung-Won Lee.

**Writing – review & editing:** Deog-Yeon Jo, Ik Chan Song.
